# Ciliated Median Raphe Cyst of Perineum Presenting as Perianal Polyp: A Case Report with Immunohistochemical Study, Review of Literature, and Pathogenesis

**DOI:** 10.1100/tsw.2006.365

**Published:** 2006-03-05

**Authors:** Jayesh Sagar, Bethani Sagar, Adam F. Patel, D.K. Shak

**Affiliations:** Royal Free Hospital, London, UK

**Keywords:** median raphe cyst, ciliated epithelium, perianal polyp, immunohistochemistry, pathogenesis

## Abstract

Median raphe cyst is a very rare, benign congenital lesion occurring mainly on the ventral aspect of the penis, but can develop anywhere in the midline between the external urethral meatus and anus. We report a case of median raphe cyst in the perineum presenting as a perianal polyp in a 65-year-old, English white male with exceptionally rare ciliated epithelium. According to our knowledge, this is the third such case of ciliated median raphe cyst in the English literature. This case, also the first case of ciliated median raphe cyst in the perineum location, focuses on pathogenesis of median raphe cyst.

## INTRODUCTION

Median raphe cyst is an uncommon, benign congenital lesion that can present anywhere in the midline between the external urethral meatus and anus. We report this rare presentation of median raphe cyst in an unusual location, the perineum, presenting as a perianal polyp in a 65-year-old, English white male with exceptionally rare ciliated epithelium. This case represents the third such case of median raphe cyst with ciliated epithelium in the English literature. According to our knowledge, this is also the first case of ciliated median raphe cyst in the perineum location. The immunohistochemical study along with review of literature, differential diagnosis, and pathogenesis of ciliated median raphe cyst are discussed here.

## CASE REPORT

The 65-year-old, English white male was referred to us by his general practitioner with complaint of a small lump near his anus for almost 4 years. It was completely asymptomatic except causing psychological embarrassment and slight discomfort. However, for the past year he was very much concerned about the lump. There was no antecedent history of trauma or infection. His medical history revealed chronic obstructive airway disease (on steroids), pneumothorax, talc pleurodesis, adhesive capsulitis of shoulder, and Dupuytren's contracture. On examination there was a 3.5- × 3-cm size mobile, nontender, cystic, well-defined lump located in the perianal region at the anterior midline (at 12 o'clock in lithotomy) position. The lesion was excised by an elliptical incision under local anesthesia and the wound closed primarily (Fig. 1). During surgical removal, the lesion was mechanically disrupted at the base, resulting in the release of cloudy pale brown fluid. The fluid was sent for culture, which yielded no pathogens. The patient developed wound infection, but recovered well with antibiotics.

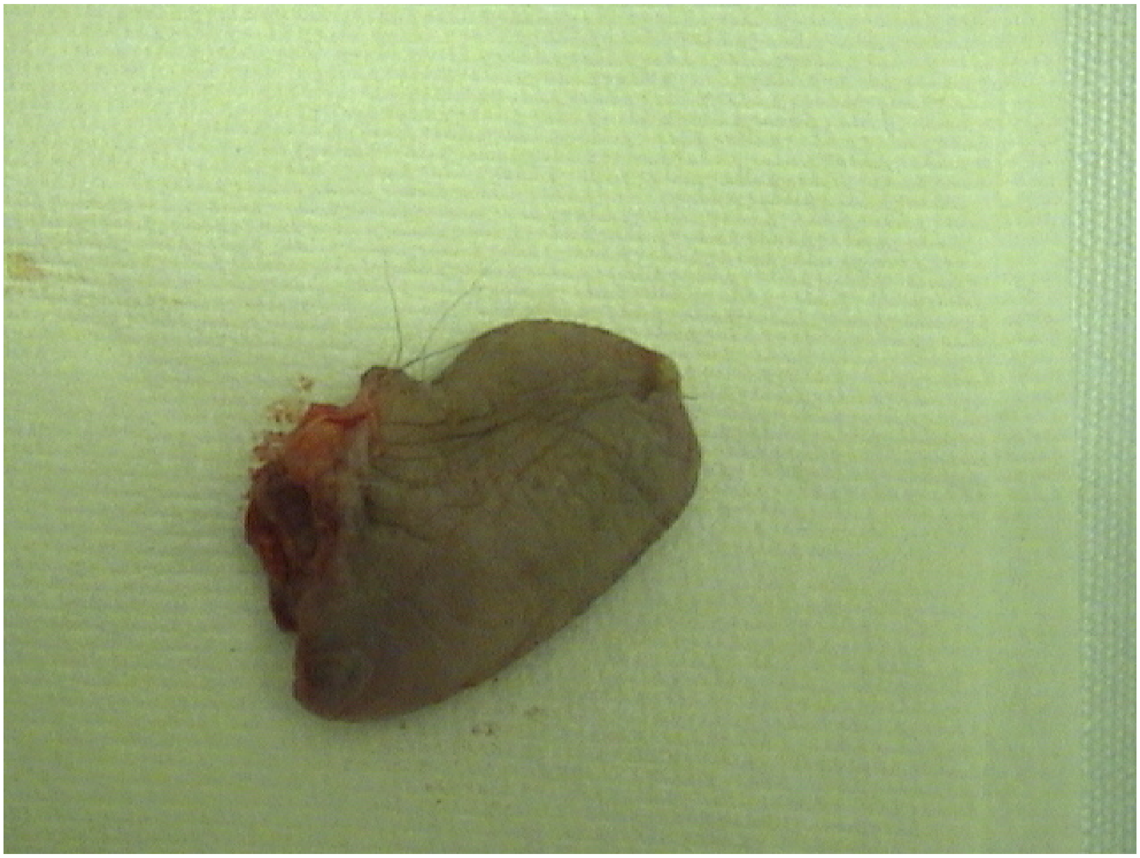

**FIGURE 1.** Macroscopic picture of excised ciliated perianal median raphe cyst.

## HISTOPATHOLOGICAL FINDINGS

The polyp was covered by normal anal skin. It contained a unilocular cyst with cloudy pale brown fluid. Most of the volume of the polyp was made up of a cyst located deep within the dermis with some surrounding smooth muscle. The cyst had no communication with the epidermis, eccrine sweat glands, hair follicles, or sebaceous glands. The deep portion of the cyst was surrounded by the skeletal muscle. No glandular structures were seen in the wall of the cyst. The cyst was lined mainly by a double layer of ciliated columnar epithelium, but in some areas the lining was up to four cells thick. At places, the epithelium was of a mature nonkeratinizing vaginal-type squamous epithelium. The epithelial lining was seen to contain scanty mucin-secreting goblet cells, but a high proportion of ciliated columnar epithelial cells with mucin secretion. There was no atypia within the epithelial lining. (Figs. 2-5).

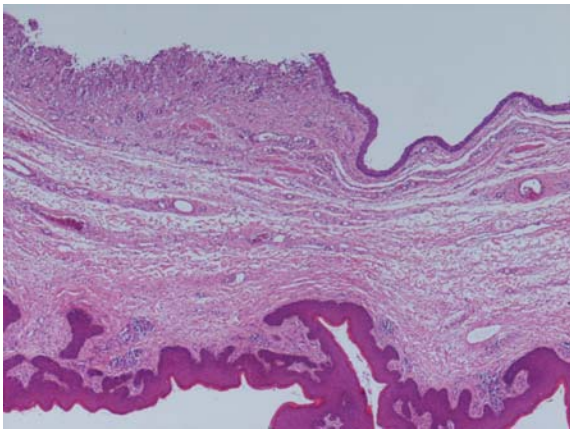

**FIGURE 2**. Epidermis covering the polyp is seen at the bottom of the picture. The columnar epithelium lining the cyst is seen at the top with an adjacent area of erosion of epithelium with foamy macrophages. Magnification ×40.

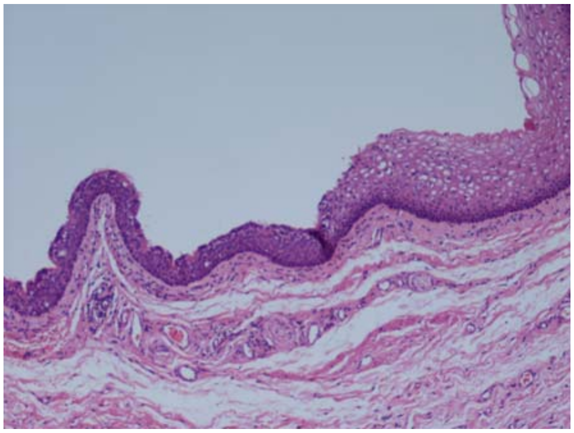

**FIGURE 3.** Cyst lining showing transition from multilayered ciliated epithelium (left) to mature nonkeratinizing squamous epithelium. Magnification ×100.

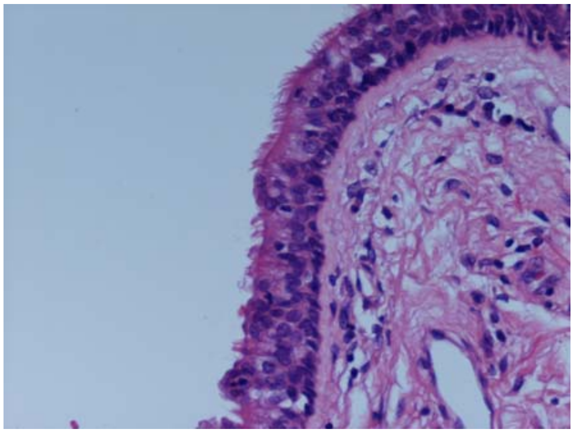

**FIGURE 4.** Multilayered ciliated columnar epithelial lining of the cyst. Magnification ×200.

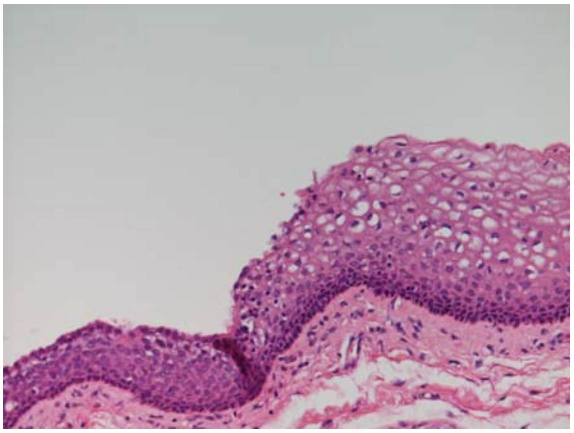

**FIGURE 5**. Area of squamous metaplasia in the cyst lining. Magnification ×200.

## IMMUNOHISTOCHEMISTRY

Immunohistochemical study showed that the epithelial lining stained positive for cytokeratin 7 (CK7) in the columnar cells, but negative in squamous cells. The cytokeratin 20 (CK20) was negative throughout. There was strong staining throughout the lining epithelium for carcinoembryonic antigen (CEA) and epithelial membrane antigen (EMA), but CA 125 stained the columnar cells only. Scattered cells were positive within both columnar and squamous areas for S100. There was no epithelial staining for the desmin or smooth muscle actin. Staining for the estrogen and progesterone receptors was negative throughout.

## DISCUSSION

Median raphe cyst is a congenital lesion occurring anywhere in the midline between the external urethral meatus and anus[1]. These kinds of cysts have been documented since 1910[2]. The other terms used for the median raphe cyst include the mucoid cyst of the penile skin, the genitoperineal cyst of the median raphe, and the parameatal cyst[3]. The usual manifestations of this cyst are in the first 3 decades of life[4,5], either due to secondary infection or due to cosmetic and psychological disfigurement[6]. Usually, the onset of this cyst is gradual, although the rapidly growing cysts have been reported in literature[7,8], one associated with postcoital appearance[7].

Median raphe cyst is an embryological developmental defect of the male genital apparatus[9]. Male genital differentiation begins after the tenth week of the embryological life due to androgenic stimulation. Subsequently, the urethral groove of the endodermal origin elongates parallel to the growth of the penis. The urethra forms at around the third month, largely as a result of the fusion of the urethral folds enveloping the urethral groove. Its most distal portion develops during the fourth month from a group of cells of the ectodermal origin in the glans tip. These cells in turn canalize to connect with the rest of the urethra[1].

The diagnosis of median raphe cyst is difficult, but needs to be differentiated from other conditions such as epidermal cyst, steatocystoma, glomus tumor, dermoid cyst, urethral diverticulum, and pilonidal cyst when it presents in the penile (most common site) and scrotal region[1,3]. Presentation of median raphe cyst in the elderly in the anterior perianal region is exceptional, especially presenting as a perianal polyp[4]. It also emphasizes inclusion of the median raphe cyst in the differential diagnosis of perianal polyp along with other conditions, such as hypertrophied papilla, viral warts, hemorrhoids, and a number of inflammatory and neoplastic conditions[4]. One should also consider the diagnosis of cutaneous ciliated cyst and tail hindgut cyst in such cases[10]. Most commonly, the diagnosis of median raphe cyst is established postoperatively on histogolical and immunohistochemical studies.

The epithelial lining of median raphe cyst includes columnar stratified, pseudostratified, or squamous cells, correlating with histology in different portions of male urethra[11]. The distinctive feature of this case is presence of ciliated cells in epithelium, which represents the third such case in the English literature. This case also represents the eldest of such patients; the previously reported cases of ciliated median raphe cyst being 24 and 30 years old[11,12]. Ciliated epithelium is present in the specific locations such as respiratory tract, middle ear, uterus and oviducts, ependymal epithelium of central nervous system, and ductuli efferentes testis. Several cystic lesions containing ciliated epithelium have been reported on human skin in the literature[11]. Bronchogenic cysts are well-known lesions found on the chest or near the mediastinum; presence of cartilage, smooth muscle, and mucous glands being characteristic features[11]. Negative staining for smooth muscle actin along with absence of just-mentioned features excludes bronchogenic cyst as diagnosis. The possibility of tail hindgut cyst is also excluded as this lesion presents as a presacral or coccygeal mass in retrorectal region. The other important differential diagnosis is the cutaneous ciliated cyst, which was first described by Hess in 1890[10,13]. However, negative staining for characteristic estrogen and progesterone receptors in typical CCC excludes diagnosis of CCC in present case. Further, the strong mucin staining of the epithelium as in our case has not been reported in the cutaneous ciliated cyst. Positive staining for CK7 in our case supports the urothelial interpretation[3]. The CK20 antigen purified from human intestinal mucosa was not detected by monoclonal antibody test. In conclusion, the CK immunostaining pattern shown by epithelial lining in this case favors simple columnar epithelium undergoing urothelial metaplasia. The immunostaining for CEA is probably related to the dysembryogenetic cloacal nature of this cyst rather than intestinal differentiation, which was not supported by the CK immunostaining pattern. Thus our observations conclude the diagnosis of median raphe cyst of perineum.

Discussion of this case pointed out the question about pathogenesis of ciliated cells in median raphe cyst. Three different theories have been proposed for pathogenesis of median raphe cyst: (1) development from urethral remnants due to a defect in the fusion of urethral folds, (2) development of the ectopic periurethral glands of Littre' that are usually located in the rectal portion of the urethra, (3) anomalous formation of epithelial buds from the urethral columnar epithelium, followed by separation[1,2]. At present, it is difficult to define any specific hypothesis for the presence of ciliated cells in these cysts due to rarity of condition. However, recently, the presence of epithelia in the penile urethra due to metaplastic changes of the urothelium, secondary to irritation or local stimulation, has been described[14], which raised the possibility of a potential role of urothelial metaplasia in our case. The presence of squamous cells in the cyst wall along with columnar epithelium in the presented case favors the metaplastic origin of ciliated cells. The other exceptional feature of this case is that it is reported as the first case of median raphe cyst of the perineum having ciliated cells in the cyst wall (the previously two reported cases being present on the penis)[11,12] in the English literature. Fortunately, benign behavior of these cysts permits curative excision, although recurrences have been documented[2]. The other proposed alternative treatment option is incision and drainage followed by electrocoagulation[6].
